# A comparison of intrapartum interventions and adverse outcomes by parity in planned freestanding midwifery unit and alongside midwifery unit births: secondary analysis of ‘low risk’ births in the birthplace in England cohort

**DOI:** 10.1186/s12884-017-1271-2

**Published:** 2017-03-21

**Authors:** Jennifer Hollowell, Yangmei Li, Kathryn Bunch, Peter Brocklehurst

**Affiliations:** 1 National Perinatal Epidemiology Unit, Nuffield Department of Population Health, University of Oxford, Old Road Campus, Headington, Oxford, OX3 7LF UK; 2 Institute for Women’s Health, University College London, London, UK

**Keywords:** Freestanding midwifery unit, Alongside midwifery unit, Birth centre, Adverse perinatal outcomes, Adverse maternal outcomes, Caesarean section, Instrumental delivery, Planned place of birth, Straightforward vaginal birth

## Abstract

**Background:**

For low risk women, there is good evidence that planned birth in a midwifery unit is associated with a reduced risk of maternal interventions compared with planned birth in an obstetric unit. Findings from the Birthplace cohort study have been interpreted by some as suggesting a reduced risk of interventions in planned births in freestanding midwifery units (FMUs) compared with planned births in alongside midwifery units (AMUs). However, possible differences have not been robustly investigated using individual-level Birthplace data.

**Methods:**

This was a secondary analysis of data on ‘low risk’ women with singleton, term, ‘booked’ pregnancies collected in the Birthplace national prospective cohort study. We used logistic regression to compare interventions and outcomes by parity in 11,265 planned FMU births and 16,673 planned AMU births, adjusted for potential confounders, using planned AMU birth as the reference group. Outcomes considered included adverse perinatal outcomes (Birthplace primary outcome measure), instrumental delivery, intrapartum caesarean section, ‘straightforward vaginal birth’, third or fourth degree perineal trauma, blood transfusion and maternal admission for higher-level care. We used a significance level of 1% for all secondary outcomes.

**Results:**

There was no significant difference in adverse perinatal outcomes between planned AMU and FMU births. The odds of instrumental delivery were reduced in planned FMU births (nulliparous: aOR 0.63, 99% CI 0.46–0.86; multiparous: aOR 0.41, 99% CI 0.25–0.68) and the odds of having a ‘straightforward vaginal birth’ were increased in planned FMU births compared with planned AMU births (nulliparous: aOR 1.47, 99% CI 1.17–1.85; multiparous: 1.86, 99% CI 1.35–2.57). The odds of intrapartum caesarean section did not differ significantly between the two settings (nulliparous: *p* = 0.147; multiparous: *p* = 0.224). The overall pattern of findings suggested a trend towards lower intervention rates and fewer adverse maternal outcomes in planned FMU births compared with planned AMU births.

**Conclusions:**

The findings support the recommendation that ‘low risk’ women can be informed that planned birth in an FMU is associated with a lower rate of instrumental delivery and a higher rate of ‘straightforward vaginal birth’ compared with planned birth in an AMU; and that outcomes for babies do not appear to differ between FMUs and AMUs.

**Electronic supplementary material:**

The online version of this article (doi:10.1186/s12884-017-1271-2) contains supplementary material, which is available to authorized users.

## Background

In 2014, the National Institute for Health and Care Excellence (NICE) updated the intrapartum care guideline to reflect recent evidence on the benefits and risks associated with planned place of birth in different settings. The updated guideline reiterated previous guidance that birth in a midwifery-led setting was associated with a reduced risk of interventions compared with planned birth in an obstetric unit (OU) and extended the guidance to cover differences in outcomes between alongside midwifery units (AMUs) and freestanding midwifery units (FMUs):

“*The evidence suggested that women planning birth in a freestanding midwifery unit had lower rates of instrumental vaginal birth and caesarean section, and therefore higher rates of spontaneous vaginal birth, than women planning birth in an alongside midwifery unit*.” ([1], p133)

However, the evidence underpinning the NICE conclusion regarding differences in interventions between AMUs and FMUs was derived from a re-analysis by the guideline development group of published tables from the Birthplace cohort study, and because this re-analysis was based on aggregated rather than individual data, the NICE comparison of outcomes in FMUs versus AMUs had a number of methodological limitations. First, while some of the analyses were stratified by parity, they were not adjusted for other potential confounders and did not account for study design effects such as clustering. Second, the NICE re-analysis involved multiple testing at the 5% level, raising the possibility that some of the apparently significant differences in outcomes identified in the analysis might be due to chance.

The purpose of this study was to replicate the NICE analysis using individual patient data and more robust statistical methods. Specifically the study aim was to compare key perinatal and maternal outcomes in ‘low risk’ women planning birth in an FMU versus women planning birth in an AMU, stratified by parity and adjusted for potential confounders, including complicating conditions identified at the start of care in labour, using 5% and 1% levels of significance for primary and secondary outcomes respectively.

## Methods

### Participants and study data

The population for this study consisted of ‘low risk’ women in the Birthplace national prospective cohort study who planned birth in an AMU or an FMU. The Birthplace cohort study setting, participants, and study data have been described in detail elsewhere [2, 3]. Briefly, the Birthplace study collected data on 79,774 ‘low’ and ‘higher risk’ births between April 2008 and April 2010 from 142 NHS trusts, 53 FMUs, 43 AMUs and a stratified random sample of 36 OUs. Women with a singleton pregnancy were eligible for inclusion if they planned a vaginal birth and received some labour care from an NHS midwife during established labour in their planned birth settings. Women who presented in preterm labour (<37 weeks’ gestation), who were ‘unbooked’ (received no antenatal care) or experienced a stillbirth prior to the onset of labour were excluded.

Planned place of birth was defined as the woman’s intended place of birth at the start of care in labour. Women were classified as ‘low risk’ if, before the onset of labour, they were not known to have any of the medical or obstetric risk factors listed in the NICE intrapartum care guideline [4].

Maternal characteristics, medical or obstetric risk factors known prior to the onset of labour, ‘complicating conditions’ noted by the midwife at the start of care in labour (for example, prolonged rupture of membranes), intrapartum interventions and adverse outcomes were recorded on a study-specific data collection form by the midwife attending the birth. Maternal and neonatal outcomes were recorded on or after day five by the midwife attending the woman.

When data for the birth episode indicated that an adverse outcome had occurred or that the baby or mother had been admitted for higher level care, additional neonatal and maternal morbidity data were extracted from the maternal and neonatal records by Birthplace local coordinating midwives using follow-up morbidity forms.

### Outcome measures

For this analysis we considered the original Birthplace primary perinatal outcome (a composite measure designed to capture adverse perinatal outcomes that may be related to the quality of intrapartum care [2, 3]) and a range of outcome measures capturing maternal interventions and outcomes:

- Perinatal outcome: ‘Birthplace primary perinatal outcome’, a composite defined as any of: stillbirth after the start of care in labour, early neonatal death, neonatal encephalopathy, meconium aspiration syndrome, brachial plexus injury, fractured humerus or clavicle.
- Maternal interventions and outcomes: instrumental delivery (ventouse or forceps delivery); intrapartum caesarean section (CS); third or fourth degree perineal trauma; blood transfusion; admission to a higher level of care; ‘straightforward vaginal birth’, a composite measure defined as birth without intrapartum CS, instrumental delivery, third or fourth degree perineal trauma or blood transfusion. This composite measure aimed to capture birth without complications that might affect future pregnancies.
- For completeness, we additionally considered the following interventions and outcomes: epidural or spinal analgesia, augmentation with syntocinon, immersion in water for pain relief, episiotomy, active management of the third stage and ‘initiation of breastfeeding’ (baby breastfed at least once). Detailed findings for these outcome measures are reported in the Additional file 1.

### Statistical analysis

Except where indicated below, statistical methods for this study followed those in the primary Birthplace analyses [2, 3]. Logistic regression was used to calculate the odds ratios and confidence intervals for each outcome. As in previous analyses we adjusted for maternal age, ethnic group, understanding of English, marital or partner status, body mass index (BMI) in pregnancy, index of multiple deprivation (IMD) score, parity (where appropriate), and gestational age at birth. In this study we additionally adjusted for complicating conditions identified at the start of care in labour, which are associated with an increased risk of transfer [5]. Analyses were stratified by parity. The Wald test was used to test for an interaction between planned place of birth and parity. For each outcome, we calculated the number of events, the weighted incidence with confidence intervals (CIs), an unadjusted odds ratio (OR), an adjusted odds ratio (aOR) controlling for potential confounders except complicating conditions, and for the main analyses, a fully adjusted odds ratio additionally controlling for ‘complicating conditions’ as a single composite variable. Robust variance estimation and probability weights were used for reasons explained elsewhere [2, 3].

The main methodological differences between the Birthplace primary analyses [2, 3] and the analyses presented here were as follows: analyses were conducted using the AMU as the reference group and were stratified by parity; analyses were adjusted for both maternal characteristics (as before) and the presence of complicating conditions identified by the midwife at the start of care in labour; as in the primary analysis we used 95% confidence intervals for the ‘Birthplace primary perinatal outcome’ and, because Birthplace had multiple secondary outcomes, we used 99% confidence intervals for all secondary outcomes to reduce the chances of ‘false positive’ findings due to multiple comparisons. However, we also present *p*-values for each comparison in addition to confidence intervals.

Stata version 13.1 was used for all analyses (StataCorp LP, College Station, TX, USA).

## Results

The Birthplace cohort included 27,992 eligible ‘low risk’ women planning birth in an FMU or AMU. The population for this analysis consisted of 27,938 ‘low risk’ women with known parity: 11,265 planning birth in an FMU and 16,673 planning birth in an AMU. Fifty four births were excluded because parity was unknown.

### Maternal characteristics of the study sample

Table 1 shows the characteristics of ‘low risk’ women who planned FMU or AMU births by planned place of birth and parity. Amongst nulliparous women, compared with women planning to give birth in an AMU, women planning birth in an FMU were more likely to be white, have a fluent understanding of English, and live in a more socioeconomically advantaged area. There was little difference in the distribution of nulliparous women’s age, marital/partner status, BMI, gestation or baby’s birthweight. Similar differences were observed amongst multiparous women (Table 1).

**Table 1:** Characteristics of ‘low risk’ women and their babies by planned place of birth and parity

	Nulliparous				Multiparous			
	FMU		AMU		FMU		AMU	
	*n* = 5187		*n* = 8350		*n* = 6078		*n* = 8323	
	*n*	%	*n*	%	*n*	%	*n*	%
**Maternal age**								
Mean (SD)	27.0	(5.69)	26.9	(5.59)	30.3	(5.39)	29.7	(5.38)
Under 20	578	11.2	906	10.9	98	1.6	158	1.9
20-24	1243	24.0	2064	24.8	886	14.6	1414	17.0
25-29	1538	29.7	2552	30.6	1720	28.3	2442	29.4
30-34	1314	25.4	2002	24.0	1930	31.8	2572	31.0
35-39	460	8.9	755	9.1	1230	20.3	1472	17.7
40+	47	0.9	56	0.7	207	3.4	242	2.9
Missing	7		15		7		23	
**Ethnic group**								
White	4779	92.2	6930	83.2	5533	91.1	6523	78.5
Indian	46	0.9	266	3.2	41	0.7	243	2.9
Pakistani	57	1.1	180	2.2	107	1.8	364	4.4
Bangladeshi	42	0.8	45	0.5	105	1.7	85	1.0
Black Caribbean	24	0.5	104	1.2	24	0.4	94	1.1
Black African	38	0.7	191	2.3	56	0.9	328	3.9
Mixed	61	1.2	143	1.7	63	1.0	150	1.8
Other	138	2.7	470	5.6	146	2.4	522	6.3
Missing	2		21		3		14	
**Understanding of English**								
Fluent	5014	96.8	7633	91.8	5896	97.3	7530	90.8
Some	142	2.7	560	6.7	131	2.2	613	7.4
None	22	0.4	126	1.5	33	0.5	148	1.8
Missing	9		31		18		32	
**Marital/Partner status**								
Married/Living together	4608	89.9	7241	88.0	5821	96.7	7745	94.4
Single/Unsupported	519	10.1	985	12.0	199	3.3	461	5.6
Missing	60		124		58		117	
**Body mass index (kg/m** ^**2**^ **)**								
Mean (SD)	23.7	(3.54)	23.6	(3.66)	24.4	(3.82)	24.4	(3.85)
Not recorded	889	17.2	1432	17.2	972	16.0	1483	17.9
10-18.4	121	2.3	243	2.9	113	1.9	194	2.3
18.5-24.9	2738	52.8	4419	53.1	2858	47.1	3783	45.7
25.0-29.9	1098	21.2	1713	20.6	1550	25.5	2071	25.0
30.0-35.0	336	6.5	521	6.3	575	9.5	748	9.0
Missing	5		22		10		44	
**Index of Multiple Deprivation (IMD) quintiles**								
1st Least deprived	1090	21.1	1241	14.9	1405	23.2	1293	15.6
2nd	1180	22.8	1357	16.3	1399	23.1	1281	15.4
3rd	1094	21.2	1687	20.3	1206	19.9	1548	18.7
4th	965	18.7	1984	23.8	1111	18.3	1860	22.4
5th Most deprived	843	16.3	2058	24.7	941	15.5	2316	27.9
Missing	15		23		16		25	
**Previous pregnancies > =24 completed weeks**								
0 Nulliparous	5187	100.0	8350	100.0	N/A	N/A	N/A	N/A
1 previous	N/A	N/A	N/A	N/A	3913	64.4	5621	67.5
2 previous	N/A	N/A	N/A	N/A	1513	24.9	1933	23.2
3+ previous	N/A	N/A	N/A	N/A	652	10.7	769	9.2
**Gestation (completed weeks)**								
Mean (SD)	39.8	(1.1)	39.7	(1.1)	39.8	(1.1)	39.7	(1.1)
37	149	2.9	257	3.1	165	2.7	216	2.6
38	473	9.1	798	9.6	505	8.3	766	9.2
39	1155	22.3	1995	24.0	1512	24.9	2130	25.7
40	1965	38.0	3178	38.2	2392	39.4	3302	39.8
41	1379	26.7	1982	23.8	1439	23.7	1814	21.8
42-44	51	1.0	119	1.4	57	0.9	76	0.9
Missing	15		21		8		19	
**Birthweight (grams)**								
Mean (SD)	3415	(420.0)	3405	(423.2)	3549	(439.3)	3519	(441.7)
1000-2499 g	57	1.1	109	1.3	43	0.7	50	0.6
2500-2999 g	725	14.0	1237	14.9	597	9.8	897	10.8
3000-3499 g	2265	43.7	3581	43.0	2163	35.6	3171	38.2
3500-3999 g	1676	32.3	2675	32.1	2342	38.6	3001	36.1
4000-4499 g	417	8.0	665	8.0	828	13.6	1034	12.5
4500-7500 g	46	0.9	56	0.7	100	1.6	149	1.8
Missing	1		27		5		21	

*SD* standard deviation

Data in bold emphasized the main headings and sub-headings

Nulliparous women were slightly more likely than multiparous women to have complicating conditions noted by the midwife at the start of care in labour, but in both groups (nulliparous and multiparous) the proportion of women with complicating conditions at the start of care in labour was similar in the two settings (Table 2).

**Table 2:** Complicating conditions identified at the start of care in labour in ‘low risk’ women by planned place of birth and parity

	Nulliparous				Multiparous			
	FMU		AMU		FMU		AMU	
	*n* = 5187		*n* = 8350		*n* = 6078		*n* = 8323	
	*n*	%	*n*	%	*n*	%	*n*	%
Prolonged rupture of membranes (>18 h)	143	2.8	260	3.1	87	1.4	122	1.5
Meconium stained liquor	77	1.5	134	1.6	63	1.0	99	1.2
Proteinuria (1+ or more)	75	1.4	227	2.7	35	0.6	142	1.7
Hypertension	46	0.9	77	0.9	31	0.5	36	0.4
Abnormal vaginal bleeding	12	0.2	29	0.3	10	0.2	8	0.1
Non-cephalic presentation	14	0.3	18	0.2	10	0.2	11	0.1
Abnormal fetal heart rate	37	0.7	40	0.5	15	0.2	25	0.3
Other	9	0.2	8	0.1	8	0.1	9	0.1
**One or more complicating conditions**	368	7.1	723	8.7	251	4.1	431	5.2

Bold data indicates the row that show summary measure of all the rows above

### Incidence

The absolute incidence of the outcome events considered varied markedly depending on the outcome (Table 3). For example, amongst nulliparous women, the incidence of the ‘Birthplace primary perinatal outcome’ was around 0.5% (i.e. 5 events per 1000 births), while maternal outcome rates ranged from 0.2–1% for maternal admission for higher level care through to 11–16% for instrumental delivery. Around 20–30% of nulliparous women and 3–5% of multiparous women experienced a birth that was not straightforward as defined in this analysis.

**Table 3:** Incidence of interventions and adverse outcomes in ‘low risk’ women by planned place of birth and parity

	Nulliparous		Multiparous	
	FMU	AMU	FMU	AMU
**Adverse perinatal outcome**	**n/1000 (95% CI)**	**n/1000 (95% CI)**	**n/1000 (95% CI)**	**n/1000 (95% CI)**
‘Birthplace primary perinatal outcome’	4.5	4.7	2.7	2.4
	(2.8-7.1)	(3.1-7.2)	(1.6-4.6)	(1.4-4.3)
**Maternal interventions and adverse outcomes**	**% (99% CI)**	**% (99% CI)**	**% (99% CI)**	**% (99% CI)**
‘Straightforward vaginal birth’	78.8	71.5	97.0	94.6
	(75.9-81.5)	(68.1-74.7)	(96.3-97.6)	(93.3-95.6)
Instrumental delivery (ventouse or forceps)	10.8	16.3	1.1	2.5
	(8.7-13.3)	(13.9-19.1)	(0.7-1.6)	(1.9-3.3)
Intrapartum caesarean section	6.7	7.7	0.7	1.0
	(5.5-8.1)	(6.3-9.3)	(0.5-1.1)	(0.7-1.5)
Third or fourth degree perineal trauma	4.0	4.9	0.9	1.6
	(3.1-5.1)	(4.0-6.0)	(0.6-1.4)	(1.2-2.1)
Blood transfusion	0.8	1.3	0.3	0.6
	(0.5-1.1)	(0.9-1.7)	(0.2-0.6)	(0.4-0.8)
Maternal admission for higher level care	0.2	1.0	0.1	0.4
	(0.1-0.5)	(0.4-2.8)	(0.0-0.3)	(0.2-0.7)

Incidence rates are weighted to reflect each unit’s duration of participation and take the clustered nature of the data into account

Data in bold emphasized the main headings and sub-headings

### Outcomes in nulliparous women

Amongst nulliparous women, those who planned birth in an FMU had highly significantly reduced odds of instrumental delivery (10.8 vs 16.3%, aOR 0.63, 99% CI 0.46–0.86, *p* < 0.001) and highly significantly increased odds of having a ‘straightforward vaginal birth’ (78.8 vs 71.5%, aOR 1.47, 99% CI 1.17–1.85, *p* < 0.001) compared with those who planned birth in an AMU (Table 4). Although not significant at the 1% level, nulliparous women who planned birth in an FMU had reduced odds of being admitted for higher level care compared with women who planned birth in an AMU (0.2 vs 1.0%, aOR 0.28, 99% CI 0.07–1.10, *p* = 0.016). None of the other outcomes, that is the ‘Birthplace primary perinatal outcome’, intrapartum caesarean section, third or fourth degree perineal trauma and blood transfusion, differed significantly between the two settings (*p* = 0.907, 0.147, 0.129 and 0.063 respectively).

**Table 4:** Interventions and adverse outcomes by planned place of birth (FMU vs AMU) in ‘low risk’ women by parity

	Nulliparous			Multiparous		
**Perinatal outcome**	**Adjusted OR**	**95% CI**	*p* value	**Adjusted OR**	**95% CI**	*p* value
‘Birthplace primary perinatal outcome’	0.96	(0.51-1.82)	0.907	1.14	(0.52-2.50)	0.745
**Maternal outcomes**	**Adjusted OR**	**99% CI**	*p* value	**Adjusted OR**	**99% CI**	*p* value
‘Straightforward vaginal birth’	1.47	(1.17-1.85)	<0.001**	1.86	(1.35-2.57)	<0.001**
Instrumental delivery (ventouse or forceps)	0.63	(0.46-0.86)	<0.001**	0.41	(0.25-0.68)	<0.001**
Intrapartum caesarean section	0.84	(0.63-1.14)	0.147	0.75	(0.41-1.38)	0.224
Third or fourth degree perineal trauma	0.82	(0.59-1.15)	0.129	0.60	(0.36-1.00)	0.010**
Blood transfusion	0.71	(0.44-1.14)	0.063	0.56	(0.26-1.21)	0.052
Maternal admission for higher level care	0.28	(0.07-1.10)	0.016*	0.30	(0.07-1.20)	0.025*

Odds ratios (OR) are fully adjusted for maternal characteristics and complicating conditions identified at the start of care in labour

Reference group = AMU

**Significant differences at the 1% level

*Significant differences at the 5% level

Data in bold emphasized the main headings and sub-headings

Use of epidural or spinal analgesia (18.9 vs 24.4%), augmentation with syntocinon (13.9 vs 18.0%), episiotomy (16.0 vs 22.1%) and active management of the third stage (79.8 vs 87.2%) were significantly less common in nulliparous women who planned birth in an FMU vs an AMU. Breastfeeding initiations (84 vs 83.7%) and use of immersion in water for pain relief (51.9 vs. 37.1%) did not differ significantly between the two settings at the 1% level of significance (Additional file 1: Table S3).

### Outcomes in multiparous women

Amongst multiparous women, those who planned birth in an FMU had highly significantly reduced odds of instrumental delivery (1.1 vs 2.5%, aOR 0.41, 99% CI 0.25–0.68, *p* < 0.001) and of third or fourth degree perineal trauma (0.9 vs 1.6%, aOR 0.60, 99% CI 0.36–1.00, *p* = 0.010) and highly significantly increased odds of having a ‘straightforward vaginal birth’ (97 vs 94.6%, aOR 1.86, 99% CI 1.35–2.57, *p* < 0.001) compared with those who planned birth in an AMU (Table 4). Although not significant at the 1% level, multiparous women who planned birth in an FMU had reduced odds of being admitted for higher level care compared with women who planned birth in an AMU (0.1 vs 0.4%, aOR 0.30, 99%CI 0.07–1.20, *p* = 0.025). None of the other outcomes, that is the ‘Birthplace primary perinatal outcome’, intrapartum caesarean section and blood transfusion, differed significantly between the two settings (*p* = 0.745, 0.224 and 0.052 respectively).

In multiparous women most other interventions were significantly less common in multiparous women who planned birth in an FMU vs an AMU (Additional file 1: Table S3): epidural or spinal analgesia (3.5 vs. 5.9%), augmentation with syntocinon (1.4 vs. 2.4%), episiotomy (2.3 vs 3.7%) and active management of the third stage (76.2 vs. 84.6%). Immersion in water for pain relief was used significantly more often by multiparous women who planned FMU birth (40.6 vs 23.2%), Breastfeeding initiation did not differ between the settings (78.2 vs. 78.6%).

Detailed results are tabulated in full in the Additional file 1.

### Sensitivity analysis

When stratified by parity we did not find a significant difference between the two birth settings (FMU and AMU) in the odds of caesarean section, third or fourth degree perineal trauma (significant only for multiparous women) or blood transfusion. However, odds ratios were in the same direction and of broadly similar magnitude in nulliparous and multiparous women, and there was no strong evidence of heterogeneity (Wald test: intrapartum caesarean section *p* = 0.558; third or fourth degree perineal trauma *p* = 0.184; blood transfusion *p* = 0.506). We therefore conducted a *post hoc* combined analysis for these three outcomes, including all women (nulliparous and multiparous) and additionally adjusting for parity.

For caesarean section, this did not show a statistically significant reduction in the odds of intrapartum caesarean section in planned FMU births compared with planned AMU births (aOR 0.82, 99% CI 0.60–1.11, *p* = 0.093).

For third or fourth degree perineal trauma, the reduction in planned FMU births (nulliparous and multiparous combined) was not significant at the 1% level (aOR 0.76, 99% CI 0.58–1.02, *p* = 0.015).

For blood transfusion, combined analysis showed a highly significant reduction in the odds of blood transfusion in planned FMU births compared with planned AMU births (aOR 0.66, 99%CI 0.44–0.99, *p* = 0.008).

## Discussion

### Summary of key findings

There was no difference in adverse perinatal outcomes, as measured by the ‘Birthplace primary perinatal outcome’, between planned AMU and FMU births. The odds of an instrumental delivery were reduced in planned FMU births and the odds of having a ‘straightforward vaginal birth’ were increased in planned FMU births compared with planned AMU births. The odds of intrapartum caesarean section did not differ significantly between the two settings. The overall pattern of the findings suggested a trend towards lower intervention rates and fewer adverse maternal outcomes in planned FMU births compared with planned AMU births.

### Strengths and limitations

Strengths and limitation of the Birthplace cohort study are discussed more fully elsewhere [3]. In brief, strengths include the ability to compare outcomes by planned place of birth at the start of care in labour, the large sample size, the minimisation of bias through achievement of a high response rate and the absence of self-selection bias arising from non-consent, and the ability to control for a range of potential confounders. In this analysis we have additionally controlled for complicating conditions identified at the start of care in labour, such as prolonged rupture of membranes, meconium stained liquor and proteinuria.

The study has a number of limitations. First, as in all Birthplace analyses, it is possible that the use of a composite perinatal outcome measure encompassing events of varying severity may have masked important differences between settings in more serious outcomes such as stillbirth, neonatal death and neonatal encephalopathy. Second, although we were able to control for a number of potential confounders, because of the non-randomised nature of the study it remains possible that women in the two study groups may have differed in ways that we did not measure and which may be associated with differences in outcome. For example, women opting for birth in an FMU may have a different attitude towards interventions and ‘natural birth’ than women who opt for birth in a hospital with medical facilities available on site, which may in turn influence their chances of receiving some of the interventions that we studied. Related to this, because the AMU and FMU groups were ‘self-selected’ (i.e. in most instances women will have ‘chosen’ an AMU or FMU) and this was a relatively uncommon choice at the time of the study, we cannot be certain that the findings are generalisable to other groups of women who may differ from those in the study sample. Finally, the findings relate to services available during the Birthplace data collection period (2008–2010) at which time there were fewer midwifery units than today and most AMUs were relatively small. Since 2010 the number of FMUs has remained relatively static but the number of AMUs has increased and the characteristics of these units (size, staffing, and admission criteria) may well have changed [6]. The generalisability of these findings to current models of service provision, clinical practice and to current users of midwifery-led services is unknown. These issues, and the need to undertake monitoring and evaluation of current services, are discussed more fully elsewhere [5].

### Interpretation

The findings of this study are broadly consistent with the unadjusted analyses conducted as part of the NICE evidence review for the 2014 intrapartum care guideline [1] and support their conclusions that perinatal outcomes are similar in the two settings and women who plan birth in an FMU are more likely to experience a spontaneous vaginal birth than women who plan birth in an AMU. Our analyses also confirmed a reduction in serious perineal trauma in women who planned birth in an FMU compared with women who planned birth in an AMU but we cannot rule out the possibility that differences could be partly attributable to different levels of ascertainment in births planned in the two settings.

We did not observe a statistically significant reduction in intrapartum caesarean section for either nulliparous or multiparous women, or overall, so our analysis does not confirm the statistically significant reduction (unadjusted RR 0.82, 95%CI 0.73–0.93) found by NICE in their analysis of aggregated data [1]. However, although we did not find a statistically significant reduction in the odds of caesarean section in planned FMU births (*p* = 0.093), the observed direction of effect (odds ratio 0.82) was not inconsistent with a possible reduction in caesarean section rates in planned FMU births.

Our results show a statistically significant reduction in instrumental delivery in births planned in an FMU compared with an AMU (10.8 vs 16.3% in nulliparous women, and 1.1 vs 2.5% in multiparous women). A number of factors might explain this, including possible differences in labour management, easier access to epidurals in births planned in an AMU (which increase the risk of instrumental delivery [7]) and possibly a higher threshold for transfer for failure to progress in the second stage of labour in births planned in an FMU (since ambulance transfer is required). However, it is also possible that women who opt for birth in an AMU differ in their attitudes towards medical interventions or in other attributes that may influence outcomes and differences in provider factors such as staff seniority and experience, and organisational culture may also play a part.

We did not find that adverse perinatal outcomes differed significantly between the two settings and for both nulliparous and multiparous women the odds of the ‘Birthplace primary perinatal outcome’ were close to one. As noted above, we cannot rule out a difference in serious adverse perinatal outcomes.

## Conclusions

Our analysis confirms that ‘low risk’ women who planned birth in an FMU had lower rates of instrumental delivery and higher rates of straightforward vaginal birth compared with women who planned birth in an AMU; and that outcomes for babies did not appear to differ between births planned in FMUs and AMUs. In general, women who planned birth in an FMU tended to experience lower intervention rates than women who planned birth in an AMU.

## Additional file

Additional file 1: Table S1. Adverse perinatal outcome by planned place of birth and parity. **Table S2.** Maternal interventions and adverse outcomes by planned place of birth and parity. **Table S3.** Additional maternal interventions and outcomes. (DOCX 33 kb)

## Abbreviations

AMU: Alongside midwifery unit
aOR: Adjusted odds ratio
BMI: Body Mass Index
CIs: Confidence intervals
CS: Caesarean section
FMU: Freestanding midwifery unit
IMD: Index of multiple deprivation
NHS: National Health Service
NICE: National Institute for Health and Care Excellence
OU: Obstetric unit
