# Supplementary material for: A comparison of intrapartum interventions and adverse outcomes by parity in planned freestanding midwifery unit and alongside midwifery unit births: secondary analysis of ‘low risk’ births in the birthplace in England cohort
Source: BMC Pregnancy Childbirth. 2017 Mar 21;17:95. doi: 10.1186/s12884-017-1271-2 (PMC5359981; doi:10.1186/s12884-017-1271-2)
Supplement: Additional file 1: Table S1. — Adverse perinatal outcome by planned place of birth and parity. Table S2. Maternal interventions and adverse outcomes by planned place of birth and parity. Table S3. Additional maternal interventions and outcomes. (DOCX 33 kb) [file 12884_2017_1271_MOESM1_ESM.docx]

# **Supplementary tables: S1-S3**

**Table S1: Adverse perinatal outcome by planned place of birth and parity**

| Planned place of Birth | | Events | Births | Incidence | | Unadjusted | | Adjusted (1) | | Adjusted (2) | |  |
| --- | --- | --- | --- | --- | --- | --- | --- | --- | --- | --- | --- | --- |
|  |  | n | n | %* | (95% CI) | OR | (95% CI) | aOR | (95% CI) | aOR | (95% CI) | p value^+^ |
| **‘Birthplace primary perinatal outcome’** | | | |  |  |  |  |  |  |  |  |  |
|  | **Nulliparous** |  |  |  |  |  | |  | |  | |  |
|  | FMU | 24 | 5158 | 4.5 | (2.8-7.1) | 0.95 | (0.51-1.77) | 0.94 | (0.51-1.76) | 0.96 | (0.51-1.82) | 0.907 |
|  | AMU | 38 | 8256 | 4.7 | (3.1-7.2) | 1 | - | 1 | - | 1 | - |  |
|  | **Multiparous** |  |  |  |  |  | |  | |  | |  |
|  | FMU | 17 | 6025 | 2.7 | (1.6-4.6) | 1.13 | (0.52-2.46) | 1.13 | (0.52-2.47) | 1.14 | (0.52-2.50) | 0.745 |
|  | AMU | 20 | 8234 | 2.4 | (1.4-4.3) | 1 | - | 1 | - | 1 | - |  |
| (1) Adjusted for maternal characteristics (age, ethnic group, understanding of English, marital/partner status, BMI, index of multiple deprivation quintile, previous pregnancies >=24 weeks, and gestation (completed weeks) | | | | | | | | | | | | |
| (2) Adjusted for maternal characteristics (as above) and complicating conditions identified at the start of care in labour | | | | | | | | | | | | |
| * Weighted; ^+^ Fully adjusted model | | | | | | | | | | | | |

**Table S2: Maternal interventions and adverse outcomes by planned place of birth and parity**

| Planned place of Birth | | Events | Births | Incidence | | Unadjusted | | Adjusted (1) | | Adjusted (2) | |  |
| --- | --- | --- | --- | --- | --- | --- | --- | --- | --- | --- | --- | --- |
|  |  | n | n | %* | (99% CI) | OR | (99% CI) | aOR | (99% CI) | aOR | (99% CI) | p value^+^ |
| **‘Straightforward vaginal birth’** | | |  |  |  |  |  |  |  |  |  |  |
|  | **Nulliparous** |  |  |  |  |  | |  | |  | |  |
|  | FMU | 4016 | 5169 | 78.8 | (75.9-81.5) | 1.48 | (1.17-1.87) | 1.49 | (1.18-1.89) | 1.47 | (1.17-1.85) | 0.000 |
|  | AMU | 5948 | 8247 | 71.5 | (68.1-74.7) | 1 | - | 1 | - | 1 | - |  |
|  | **Multiparous** |  |  |  |  |  | |  | |  | |  |
|  | FMU | 5841 | 6032 | 97.0 | (96.3-97.6) | 1.86 | (1.35-2.56) | 1.88 | (1.35-2.61) | 1.86 | (1.35-2.57) | 0.000 |
|  | AMU | 7790 | 8224 | 94.6 | (93.3-95.6) | 1 | - | 1 | - | 1 | - |  |
| **Instrumental delivery** | | |  |  |  |  |  |  |  |  |  |  |
|  | **Nulliparous** |  |  |  |  |  | |  | |  | |  |
|  | FMU | 613 | 5186 | 10.8 | (8.7-13.3) | 0.62 | (0.46-0.84) | 0.62 | (0.46-0.86) | 0.63 | (0.46-0.86) | 0.000 |
|  | AMU | 1327 | 8336 | 16.3 | (13.9-19.1) | 1 | - | 1 | - | 1 | - |  |
|  | **Multiparous** |  |  |  |  |  | |  | |  | |  |
|  | FMU | 71 | 6078 | 1.1 | (0.7-1.6) | 0.43 | (0.26-0.70) | 0.41 | (0.25-0.67) | 0.41 | (0.25-0.68) | 0.000 |
|  | AMU | 193 | 8317 | 2.5 | (1.9-3.3) | 1 | - | 1 | - | 1 | - |  |
| **Intrapartum caesarean section** | | |  |  |  |  |  |  |  |  |  |  |
|  | **Nulliparous** |  |  |  |  |  | |  | |  | |  |
|  | FMU | 357 | 5186 | 6.7 | (5.5-8.1) | 0.86 | (0.64-1.15) | 0.83 | (0.61-1.14) | 0.84 | (0.63-1.14) | 0.147 |
|  | AMU | 637 | 8336 | 7.7 | (6.3-9.3) | 1 | - | 1 | - | 1 | - |  |
|  | **Multiparous** |  |  |  |  |  | |  | |  | |  |
|  | FMU | 46 | 6078 | 0.7 | (0.5-1.1) | 0.71 | (0.39-1.29) | 0.74 | (0.40-1.40) | 0.75 | (0.41-1.38) | 0.224 |
|  | AMU | 88 | 8317 | 1.0 | (0.7-1.5) | 1 | - | 1 | - | 1 | - |  |

*/continued*

**Table S2: Maternal interventions and adverse outcomes by planned place of birth and parity *(continued)***

| Planned place of Birth | | Events | Births | Incidence | | Unadjusted | | Adjusted (1) | | Adjusted (2) | |  |
| --- | --- | --- | --- | --- | --- | --- | --- | --- | --- | --- | --- | --- |
|  |  | n | n | %* | (99% CI) | OR | (99% CI) | aOR | (99% CI) | aOR | (99% CI) | p value^+^ |
| **Third or fourth degree perineal trauma** | | | |  |  |  |  |  |  |  |  |  |
|  | **Nulliparous** |  |  |  |  |  | |  | |  | |  |
|  | FMU | 206 | 5177 | 4.0 | (3.1-5.1) | 0.82 | (0.58-1.14) | 0.82 | (0.59-1.15) | 0.82 | (0.59-1.15) | 0.129 |
|  | AMU | 405 | 8322 | 4.9 | (4.0-6.0) | 1 | - | 1 | - | 1 | - |  |
|  | **Multiparous** |  |  |  |  |  | |  | |  | |  |
|  | FMU | 52 | 6068 | 0.9 | (0.6-1.4) | 0.57 | (0.34-0.96) | 0.60 | (0.36-1.00) | 0.60 | (0.36-1.00) | 0.010 |
|  | AMU | 129 | 8295 | 1.6 | 1.2-2.1 | 1 | - | 1 | - | 1 | - |  |
| **Blood transfusion** | |  |  |  |  |  |  |  |  |  |  |  |
|  | **Nulliparous** |  |  |  |  |  | |  | |  | |  |
|  | FMU | 42 | 5173 | 0.8 | (0.5-1.1) | 0.60 | (0.37-0.98) | 0.70 | (0.43-1.12) | 0.71 | (0.44-1.14) | 0.063 |
|  | AMU | 93 | 8262 | 1.3 | (0.9-1.7) | 1 | - | 1 | - | 1 | - |  |
|  | **Multiparous** |  |  |  |  |  | |  | |  | |  |
|  | FMU | 25 | 6040 | 0.3 | (0.2-0.6) | 0.54 | (0.26-1.13) | 0.56 | (0.26-1.20) | 0.56 | (0.26-1.21) | 0.052 |
|  | AMU | 43 | 8250 | 0.6 | (0.4-0.8) | 1 | - | 1 | - | 1 | - |  |
| **Maternal admission for higher level care** | | | |  |  |  |  |  |  |  |  |  |
|  | **Nulliparous** |  |  |  |  |  | |  | |  | |  |
|  | FMU | 15 | 5187 | 0.2 | (0.1-0.5) | 0.24 | (0.07-0.88) | 0.28 | (0.07-1.08) | 0.28 | (0.07-1.10) | 0.016 |
|  | AMU | 51 | 8350 | 1.0 | (0.4-2.8) | 1 | - | 1 | - | 1 | - |  |
|  | **Multiparous** |  |  |  |  |  | |  | |  | |  |
|  | FMU | 9 | 6078 | 0.1 | (0.0-0.3) | 0.26 | (0.07-1.00) | 0.30 | (0.07-1.19) | 0.30 | (0.07-1.20) | 0.025 |
|  | AMU | 31 | 8323 | 0.4 | (0.2-0.7) | 1 | - | 1 | - | 1 | - |  |
| (1) Adjusted for maternal characteristics (age, ethnic group, understanding of English, marital/partner status, BMI, index of multiple deprivation quintile, previous pregnancies >=24 weeks, and gestation (completed weeks) | | | | | | | | | | | | |
| (2) Adjusted for maternal characteristics (as above) and complicating conditions identified at the start of care in labour | | | | | | | | | | | | |
| * Weighted; ^+^ Fully adjusted model | | | | | | | | | | | | |

**Table S3: Additional maternal interventions and outcomes**

| Planned place of Birth | | Events | Births | Incidence | | Unadjusted | | Adjusted (1) | | Adjusted (2) | |  |
| --- | --- | --- | --- | --- | --- | --- | --- | --- | --- | --- | --- | --- |
|  |  | n | n | %* | (99% CI) | OR | (99% CI) | aOR | (99% CI) | aOR | (99% CI) | p value^+^ |
| **Epidural or spinal analgesia** | | |  |  |  |  |  |  |  |  |  |  |
|  | **Nulliparous** |  |  |  |  |  | |  | |  | |  |
|  | FMU | 1021 | 5168 | 18.9 | (16.5-21.6) | 0.72 | (0.57-0.91) | 0.71 | (0.56-0.90) | 0.72 | (0.57-0.90) | 0.000 |
|  | AMU | 1987 | 8320 | 24.4 | (21.5-27.7) | 1 | - | 1 | - | 1 | - |  |
|  | **Multiparous** |  |  |  |  |  | |  | |  | |  |
|  | FMU | 224 | 6068 | 3.5 | (2.8-4.5) | 0.59 | (0.42-0.82) | 0.56 | (0.40-0.77) | 0.56 | (0.41-0.77) | 0.000 |
|  | AMU | 472 | 8305 | 5.9 | (4.8-7.1) | 1 | - | 1 | - | 1 | - |  |
| **Augmentation with Syntocinon** | | |  |  |  |  |  |  |  |  |  |  |
|  | **Nulliparous** |  |  |  |  |  | |  | |  | |  |
|  | FMU | 778 | 5158 | 13.9 | (11.8-16.3) | 0.73 | (0.58-0.93) | 0.72 | (0.56-0.92) | 0.73 | (0.57-0.94) | 0.001 |
|  | AMU | 1507 | 8318 | 18.0 | (15.9-20.3) | 1 | - | 1 | - | 1 | - |  |
|  | **Multiparous** |  |  |  |  |  | |  | |  | |  |
|  | FMU | 96 | 6065 | 1.4 | (0.9-2.1) | 0.56 | (0.33-0.96) | 0.55 | (0.32-0.94) | 0.56 | (0.33-0.95) | 0.005 |
|  | AMU | 199 | 8305 | 2.4 | (1.8-3.3) | 1 | - | 1 | - | 1 | - |  |
| **Immersion in water for pain relief** | | |  |  |  |  |  |  |  |  |  |  |
|  | **Nulliparous** |  |  |  |  |  | |  | |  | |  |
|  | FMU | 2726 | 5178 | 51.9 | (41.2-62.5) | 1.84 | (1.04-3.24) | 1.68 | (0.96-2.95) | 1.67 | (0.95-2.91) | 0.018 |
|  | AMU | 3077 | 8337 | 37.1 | (29.0-45.9) | 1 | - | 1 | - | 1 | - |  |
|  | **Multiparous** |  |  |  |  |  | |  | |  | |  |
|  | FMU | 2520 | 6075 | 40.6 | (30.7-51.2) | 2.26 | (1.29-3.94) | 1.98 | (1.15-3.42) | 1.98 | (1.15-3.42) | 0.001 |
|  | AMU | 1975 | 8319 | 23.2 | (17.5-30.1) | 1 | - | 1 | - | 1 | - |  |
| */continued* | | | | | | | | | | | | |

**Table S3: Additional maternal interventions and outcomes (continued**)

| Planned place of Birth | | | | Events | Births | | Incidence | | | | | Unadjusted | | | Adjusted (1) | | | | Adjusted (2) | |  |
| --- | --- | --- | --- | --- | --- | --- | --- | --- | --- | --- | --- | --- | --- | --- | --- | --- | --- | --- | --- | --- | --- |
|  |  |  |  | n | n | | %* | | (99% CI) | | | OR | (99% CI) | | aOR | (99% CI) | | | aOR | (99% CI) | p value+ |
| **Episiotomy** | | | | |  | |  | |  | | |  |  | |  |  | | |  |  |  |
|  | | **Nulliparous** | |  |  | |  | |  | | |  | | |  | | | |  | |  |
|  | | FMU | | 855 | 5183 | | 16.0 | | (13.3-19.1) | | | 0.67 | (0.51-0.88) | | 0.67 | (0.50-0.89) | | | 0.67 | (0.51-0.89) | 0.000 |
|  | | AMU | | 1804 | 8337 | | 22.1 | | (19.3-25.2) | | | 1 | - | | 1 | - | | | 1 | - |  |
|  | | **Multiparous** | |  |  | |  | |  | | |  | | |  | | | |  | |  |
|  | | FMU | | 137 | 6076 | | 2.3 | | (1.8-3.0) | | | 0.61 | (0.43-0.87) | | 0.58 | (0.40-0.84) | | | 0.58 | (0.41-0.84) | 0.000 |
|  | | AMU | | 287 | 8315 | | 3.7 | | (3.0-4.6) | | | 1 | - | | 1 | - | | | 1 | - |  |
| **Active management of the third stage of labour** | | | | |  | |  | |  | | |  |  | |  |  | | |  |  |  |
|  | | **Nulliparous** | |  |  | |  | |  | | |  | | |  | | | |  | |  |
|  | | FMU | | 4127 | 5179 | | 79.8 | | (72.4-85.5) | | | 0.58 | (0.33-1.01) | | 0.55 | (0.32-0.95) | | | 0.55 | (0.32-0.95) | 0.005 |
|  | | AMU | | 7191 | 8335 | | 87.2 | | (82.4-90.8) | | | 1 | - | | 1 | - | | | 1 | - |  |
|  | | **Multiparous** | |  |  | |  | |  | | |  | | |  | | | |  | |  |
|  | | FMU | | 4561 | 6076 | | 76.2 | | (67.5-83.2) | | | 0.58 | (0.33-1.04) | | 0.57 | (0.32-1.00) | | | 0.57 | (0.32-1.00) | 0.010 |
|  | | AMU | | 6904 | 8320 | | 84.6 | | (79.1-88.9) | | | 1 | - | | 1 | - | | | 1 | - |  |
| **Breastfed at least once** | | | | | |  | |  | |  |  | |  |  | | |  |  | |  |  |
|  | **Nulliparous** | |  | | |  | |  | |  |  | | |  | | | |  | | |  |
|  | FMU | | 4318 | | | 5148 | | 84.0 | | (78.9-88.1) | 1.02 | | (0.53-1.96) | 1.08 | | | (0.66-1.78) | 1.08 | | (0.66-1.76) | 0.695 |
|  | AMU | | 6799 | | | 8269 | | 83.7 | | (74.8-89.9) | 1 | | - | 1 | | | - | 1 | | - |  |
|  | **Multiparous** | |  | | |  | |  | |  |  | | |  | | | |  | | |  |
|  | FMU | | 4726 | | | 6026 | | 78.2 | | (72.2-83.3) | 0.98 | | (0.55-1.74) | 1.00 | | | (0.64-1.57) | 1.00 | | (0.64-1.57) | 0.994 |
|  | AMU | | 6362 | | | 8258 | | 78.6 | | (69.5-85.5) | 1 | | - | 1 | | | - | 1 | | - |  |
| (1) Adjusted for maternal characteristics (age, ethnic group, understanding of English, marital/partner status, BMI, index of multiple deprivation quintile, previous pregnancies >=24 weeks, and gestation (completed weeks) | | | | | | | | | | | | | | | | | | | | | |
| (2) Adjusted for maternal characteristics (as above) and complicating conditions at start of care in labour | | | | | | | | | | | | | | | | | | | | | |
| * Weighted; + Fully adjusted model | | | | | | | | | | | | | | | | | | | | | |
